# Urinary Tract Infections and Associated Factors among Patients with Indwelling Urinary Catheters Attending Bugando Medical Centre a Tertiary Hospital in Northwestern Tanzania

**DOI:** 10.3390/microorganisms10020473

**Published:** 2022-02-21

**Authors:** Asteria L. M. Ndomba, Rose M. Laisser, Vitus Silago, Benson R. Kidenya, Joseph Mwanga, Jeremiah Seni, Stephen E. Mshana

**Affiliations:** 1 Archbishop Anthony Mayala School of Nursing, Catholic University of Health and Allied Sciences, Mwanza P.O. Box 1464, Tanzania; roselaisser.rl@gmail.com; 2 Department of Microbiology and Immunology, Weill Bugando School of Medicine, Catholic University of Health and Allied Sciences, Mwanza P.O. Box 1464, Tanzania; vsilago@bugando.ac.tz (V.S.); senijj80@gmail.com (J.S.); stephen72mshana@gmail.com (S.E.M.); 3 Department of Biochemistry and Molecular Biology, Weill Bugando School of Medicine, Catholic University of Health and Allied Sciences, Mwanza P.O. Box 1464, Tanzania; benkidenya@gmail.com; 4 Department of Biostatistics, Epidemiology and Behavioral Sciences, Weill Bugando School of Medicine, Catholic University of Health and Allied Sciences, Mwanza P.O. Box 1464, Tanzania; jrmwanga@yahoo.co.uk

**Keywords:** Bugando Medical Centre, *Escherichia coli*, indwelling urinary catheterization, *Klebsiella* species, urinary tract infections

## Abstract

Complications of indwelling urinary catheterization (IUC) are associated with significant morbidity and mortality, thus affecting patient’s well-being. Understanding the magnitude and factors associated with complications is crucial in designing appropriate preventive strategies. A cross-sectional study was conducted at Bugando Medical Centre, involving patients with long-term and short-term IUC from December 2016 to September 2017. The data were analyzed by STATA 13.0. Catheter-associated urinary tract infection (CA-UTI) was the leading (56.8%; 250/440) complication among patients with IUC. Gram-negative bacteria were predominantly isolated (98.1%, 252/257), whereas *E. coli* (30.7%, 79/257) and *Klebsiella* spp. (29.6%, 76/257) were the leading pathogens. CA-UTI was significantly higher among out-patients than in-patients (82.2% v 35.3%, *p* < 0.001). Older age (OR: 1.3, (95%CI: 1.1–1.5), *p* < 0.001), level of education (OR: 1.8, (95%CI: 1.1–3.1), *p* = 0.029) and catheter duration of ≥6 weeks (OR: 2.43, (95%CI: 1.1–5.5), *p* = 0.031) independently predicted CA-UTI among outpatients, while female gender (OR: 2.1, (95%CI: 1.2–3.7), *p* = 0.014), catheter bags not freely hanging (OR: 0.4, (95%CI: 0.2–0.7), *p* = 0.002) and residing outside Mwanza region (OR: 0.4, (95%CI: 0.2–0.6), *p* < 0.001) predicted CA-UTI among in-patients. CA-UTI is the common complication among patients with IUC, significantly higher in out-patients than in-patients. We recommend involving patients and carers in infection prevention and control measures in out-patients living with IUC.

## 1. Introduction

Complications of indwelling urinary catheterization (IUC) affect patients’ well-being, mostly physically, psychologically and socially [1]. Common complications documented in several studies include catheter blockages, leakage, urethral hemorrhage, urethritis, bladder spasms, bladder calculi, vesicoureteral reflux and over the years bladder cancer [2,3]. However, the most frequent and serious complication is the catheter-associated urinary tract infection (CA-UTI), which is associated with reduced quality of life, increased risk of hospitalization and increased mortality [4].

CA-UTI accounts for up to 25% of healthcare-associated infections and is the most common complication for both patients with short- and long-term IUCs [5,6,7]. Gram-negative bacteria (GNB), notably *Escherichia coli*, *Klebsiella* species (i.e., *K. aerogenes)*, *Proteus mirabilis* and *Pseudomonas aeruginosa*, and Gram positive bacteria (GPB), mostly *Staphylococcus aureus* and *Enterococcus* species, are predominantly isolated causing CA-UTI [8,9,10,11]. Although *E.coli* and *Klebsiella* spp. are the most frequently involved bacteria, and the reservoir is the patient’s own gut microbiota, the multi-drug resistant (MDR) strains are acquired elsewhere [12,13,14]. Sometimes, patients with IUC acquire these superbugs as nosocomial infections. [15]. 

Increased patient’s age and duration of catheterization, severe illness, obesity, immunocompromised status, frequent clinics or hospital visits and hospitalization are among the factors reported to increase the risks of CA-UTI [2,16]. Studies have reported that IUC is more common among older women and men, as well as being more common in low- and middle-income countries (LMICs) [17,18,19]. A recent study from Tanzania reported a prevalence of 9.6% of long-term IUC among out-patients [20]. The longer the catheter is in situ, the higher the risk of developing CA-UTI [6,7,21,22]. 

It is estimated that 8.5–10% of patients with IUC develop CA-UTI in developed countries, while as many as 42–50% of patients with IUC develop CA-UTI in LMICs [23,24,25]. The reasons for the high CA-UTI in LMIC could possibly be due to poor infection prevention practices, a poor socioeconomic status to pay for health care services, inadequate health care infrastructure and the lack of stringent policies in place, compared to developed countries that enforce the use of IUC only when indicated and removed as soon as possible [20,26,27]. In Tanzania, there is limited information on the complications associated with IUC. Therefore, to our knowledge, this is the first study to investigate CA-UTI and the associated factors among patients living with short- and long-term IUC at Bugando Medical Center (BMC), a tertiary hospital in northwestern Tanzania.

## 2. Materials and Methods

### 2.1. Study Design, Duration and Settings

This was a cross-sectional analytical study conducted between December 2016 and September 2017 at Bugando Medical Center (BMC), a tertiary hospital in northwestern Tanzania. BMC serves as the Zonal Referral Hospital for eight Lake Zone regions, namely Mwanza, Simiyu, Mara, Kagera, Shinyanga, Geita, Tabora and Kigoma, with an estimated population of 13 million people. BMC has an estimated capacity of 1000 beds. 

### 2.2. Study Population, Inclusion and Exclusion Criteria

The study population included adult patients, ≥18 years, with IUC out-patients attending urology clinics and in-patients admitted to the medical, surgical, urology, gynecology, oncology wards, and the intensive care unit at BMC, in Mwanza, Tanzania. The minimum sample size for the study was 384, calculated by using the Kish–Leslie formula (1965) using a prevalence of 50%. Long term IUC according to our study was defined as having an indwelling Foley’s urinary catheter >14 days continuously; these were the out-patients. This is supported by evidence-based guidelines for best practice in urological health care [28]. Therefore, in-patients included a population of participants with short-term IUC, while out-patients made a population of participants with long-term IUC. During the study period, about 2112 out-patients with different conditions, including those with indwelling urinary catheters, attended the urology clinics. Only 202 out-patients with a long-term IUC met the inclusion criteria and were enrolled. For in-patients, 238 patients were eligible and enrolled in the study making a total of 440 participants (202 out-patients and 238 in-patients). To ensure that the enrolled participants were not duplicated, a unique identification mark was attached to the file of each participant once enrolled. 

### 2.3. Determination and Definition of Complications

In this study, complications were determined as the presence of problems that a patient experienced after the insertion of a catheter in the bladder, such as UTI, blockage, bleeding and leakage. These complications were objectively observed and confirmed (e.g., CA-UTI) by the researchers as defined below:

*CA-UTI* was defined as a patient with symptoms (e.g., fever, urinary frequency or urgency, dysuria and suprapubic tenderness), having an indwelling urinary catheter for more than 2 days by the date of sampling and urine culture with more than 105 CFU/mL of one bacterial species [29].

*Urine blockage* was defined as the catheter that was not draining any urine into the catheter bag [30], while urine leakage was defined as the inability urine flow through the catheter that results from the buildup of the crystalline material due to biofilm formation on the luminal surfaces of the catheter tube. As a consequence, urine often leaks along the outside of the catheter and patients become incontinent [30].

*Bleeding* was defined as the passage of blood stained urine into the catheter bag, mostly caused by crystalline biofilms formed outside the catheter, leading to the irritation and trauma of the urethral mucosa leading to bleeding [31]. 

### 2.4. Data and Sample Collection

Socio-demographic (e.g., age, sex, education level and residence) and clinical related (e.g., catheter type, catheter size, and duration of catheterization) information were collected by using structured questionnaires and reviews of the patients’ medical records. About 10 mL of urine samples was collected using a sterile container from the catheter tube from each patient for quantitative culture. Briefly, the catheter tube was clamped proximally to the urethral or suprapubic opening to allow for the collection of fresh voided urine. The site of aspiration in the catheter tube was cleaned using 70% alcohol. A sterile needle and syringe was used to aspirate about 10 mL of urine sample from the catheter tube into a sterile and labeled urine container. Urine samples were sent to the Microbiology Laboratory at the Catholic University of Health and Allied Sciences (CUHAS) for processing within 2 h of collection in a cool box with ice packs maintaining a temperature ranging from 2 °C to 8 °C. 

### 2.5. Laboratory Procedures 

#### 2.5.1. Urine Culture

Urine samples were directly and quantitatively inoculated on 5% sheep blood agar (SBA; Oxoid, UK) and MacConkey agar (MCA; Oxoid, UK) plates. Plates were incubated aerobically at 37 °C for 24 h. All samples with significant growth, ≥10^5^ CFU/mL, isolates were identified to possible species level by using in-house prepared biochemical identification media (Gram stain, catalase, coagulase, DNase, novobiocin disc, bile aesculin agar, TSI agar, SIM agar, Simmons citrate agar, Christensen’s urea agar, and oxidase), as reported previously [32,33], followed by antibiotics susceptibility testing as recommended by Clinical and Laboratory Standards Institute (CLSI) guidelines [34]. 

#### 2.5.2. Antibiotics Susceptibility Testing and Phenotypic Detection of Extended Spectrum β-Lactamase Production in *E. coli* and *Klebsiella* spp.

The Kirby Bauer disc diffusion technique was used for antibiotics susceptibility testing (AST). Briefly, test bacteria were suspended in sterile physiological saline 0.85% to obtain a turbidity equivalent to 0.5 McFarland turbidity standard. Then, the surfaces of Mueller Hinton agar (MHA; Oxoid, UK) plates were swabbed to obtain even lawns and antibiotic discs were seeded within 15 min. For Gram-negative bacteria, nitrofurantoin 300 (F 300), ciprofloxacin 5 µg (CIP 5 µg), ampicillin 30 µg (AMP 30 µg), gentamicin 10 µg (CN 10 µg), amoxicillin-clavulanic acid 30 µg (AMC 30 µg), ceftriaxone 30 µg (CRO 30 µg), ceftazidime 30 µg (CAZ 30 µg), trimethoprim-sulfamethoxazole 25 µg (SXT 25 µg) and meropenem 10 µg (MEM 10 µg) were seeded, whereas for Gram positive bacteria, vancomycin 30 µg (VA 30 µg), gentamicin 10 µg (CN 10 µg), tetracycline 30 µg (TE 30 µg), erythromycin 30 µg (E 30 µg), cefoxitin 30 µg (FOX 30 µg), ciprofloxacin 5 µg (CIP 5 µg) and nitrofurantoin 300 (F 300) were seeded. All antibiotic discs used were from Oxoid, UK. Plates of MHA were incubated aerobically at 37 °C for 24 h. The zones of inhibition around the antibiotic discs were measured in millimeters and interpreted as susceptible, intermediate and resistant using Clinical and Laboratory Standards Institute (CLSI) [34]. The production of extended spectrum β-lactamase (ESBL) was tested using the CLSI disc combination method. Isolate showing an increased zone of inhibition of ≥5 mm between cephalosporin discs with clavulanic acid and without clavulanic acid was phenotypically confirmed as an ESBL producer [34]. 

### 2.6. Quality Control

*E. coli* ATCC 25922 and *S. aureus* ATCC 25923 were used as control organisms. 

### 2.7. Statistical Analysis

Quantitative data were entered into Microsoft Excel for cleaning and coding, then into STATA software version 13.0 for analysis. Descriptive statistics, such as percentages and frequencies, were used for categorical variables, whereas the median (inter quartile range (IQR)) was used for continuous variables. Univariate and multivariate logistic regression, as well as Generalized Linear Model analysis, were used to determine the factors associated with the complications. The variables that showed a significant *p*-value (<0.05) in the univariate analysis were subjected to multivariate logistic regression. 

### 2.8. Ethical Considerations 

This study was ethically cleared by the joint Catholic University of Health and Allied Sciences (CUHAS) and Bugando Medical Centre (BMC) Research Ethics and Review Committee with the ethical clearance certificate number CREC.152/2016. Permission to conduct this study was sought from the Bugando Medical Centre’s administration. Participants were requested to sign informed consent forms before being enrolled in this study. Participants’ related information were stored anonymously by using unique identifying numbers to ensure confidentiality throughout the study. 

## 3. Results

### 3.1. Socio-Demographic and Clinical Characteristics of Participants with Short-Term IUC (In-Patients) and Long-Term IUC (Out-Patients)

A total of 440 (202 with long-term IUC and 238 with short-term IUC) participants were enrolled. Out-patients with long-term IUC were older than in-patients with short-term IUC, 69 (IQR: 61–77) years vs. 46 (IQR: 30–62) years, *p* < 0.001. Significantly, more males were enrolled in the out-patients group than in-patients (96.0% vs. 55.5%, *p* < 0.001) (Table 1). The majority of participants had urethral catheterization (59.4% out-patients and 95.8% in-patients), although among those with supra-pubic catheterization, the majority were out-patients rather than in-patients (40.6% vs. 4.2%, *p* < 0.001). Among those with long-term IUC (out-patients), the majority changed catheters at least once (61.0% vs. 39.1%, *p* < 0.001). Significantly, the out-patients with long-term IUC had benign prostatic hypertrophy (BPH), compared with in-patients with short-term IUC (60.4% vs. 3.8%, *p* < 0.001). The majority of participants with IUC had no other comorbidities (65.3% out-patients and 65.1% in-patients) (Table 2). 

### 3.2. Prevalence and Pathogens Causing Catheter-Associated Urinary Tract Infection (CA-UTI) among Participants with Short-Term and Long-Term IUC

**Figure 1:**
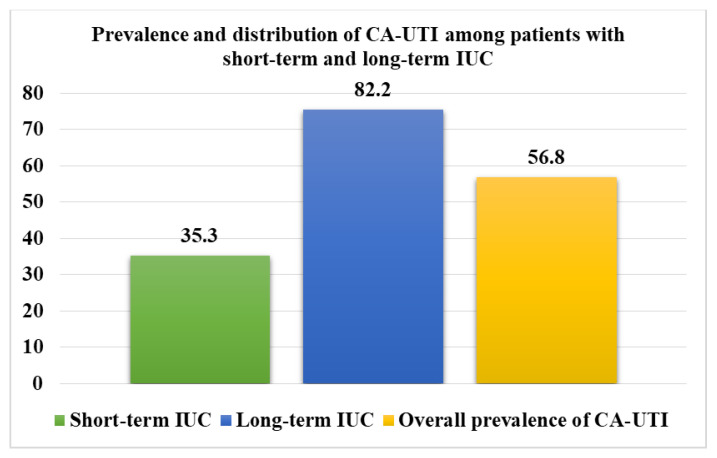
Prevalence of catheter-associated urinary tract infection (CA-UTI) among participants with short-term and long-term IUC.

**Figure 2:**
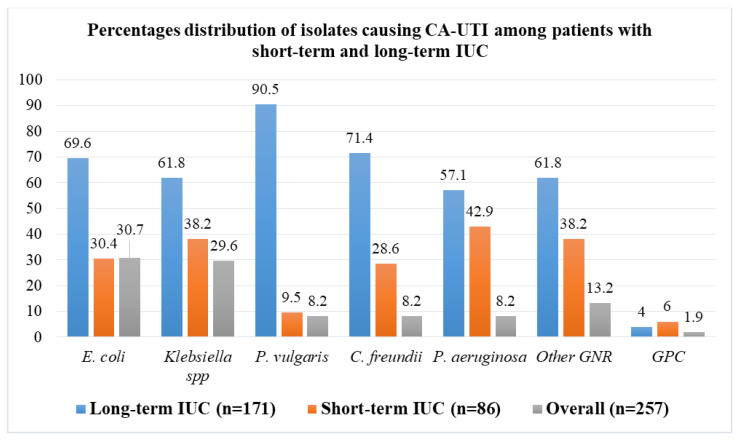
Pathogens causing catheter-associated urinary tract infection (CA-UTI) among participants with short-term and long-term IUC.

The overall prevalence of laboratory-confirmed CA-UTI among patients with IUC was 56.8% (250/440). Patients with long-term IUC had a significantly higher prevalence of CA-UTI than patients with short-term IUC (82.2% (166/202) vs. 35.3% (84/238) *p* < 0.001), (Figure 1). Seven patients had dual bacterial growth, resulting in a total of 257 isolates. Gram-negative rods (GNRs) were predominantly isolated (98.1%, 252/257), whereas *E. coli* (30.7%, 79/257) and *Klebsiella* spp. (29.6%, 76/257) were the leading causes of CA-UTI among patients with IUC. Only 1.9% (5/257) of isolates causing CA-UTI among patients with IUC at this setting were Gram-positive cocci (GPC) (Figure 2). 

### 3.3. Percentages Resistance of Bacteria Causing CA-UTI among Patients with Short-Term and Long-Term IUC

The general percentage resistance of *E. coli* causing CA-UTI among patients with short-term and long-term IUC were 94.6% ampicillin, 84.8% trimethoprim/sulfamethoxazole, 43.0% gentamicin, 50.6% ciprofloxacin, 55.4% ceftriaxone, 50.7% ceftazidime, and 35.4% nitrofurantoin, whereas the general percentage resistance of *Klebsiella* spp. causing CA-UTI among patients with short-term and long-term IUC were 100% ampicillin, 82.9% trimethoprim/sulfamethoxazole, 31.6% gentamicin, 32.9% ciprofloxacin, 47.4% ceftriaxone, 43.4% ceftazidime, and 46.1% nitrofurantoin. Moreover, other GNR exhibited a resistance of 73.1% ampicillin, 55.1% trimethoprim/sulfamethoxazole, 20.2% gentamicin, 31.3% ciprofloxacin, 33.3% ceftriaxone, 29.3% ceftazidime, and 40.4% nitrofurantoin. Generally, from this study, we observed that pathogens from in-patients were more resistant to antibiotics than pathogens from out-patients. There was no statistical significant difference of ESBL production between *E. coli* and *Klebsiella* spp. (50.6% vs. 47.4%, *p* = 0.3923) (Table 3).

### 3.4. Factors Associated with Complications among Patients with Short-Term and Long-Term IUC

**Figure 3:**
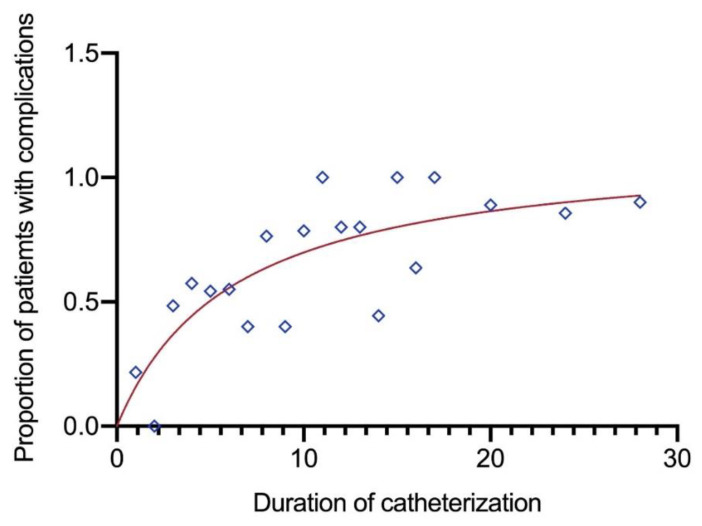
Increasing duration (days) of catheterization increases the risks of complications.

As the duration increases, the risk of complications is found to increase (OR: 3.8; 95%CI: 2.8–5.2; *p* < 0.001) (Figure 3). On logistic regression analysis, for the patients with long-term IUC, the level of education (OR: 1.8; (95%CI: 1.1–3.1); *p* = 0.029) was significantly associated with complications and catheterization of ≥6 weeks, increases the risk of complications (OR: 1.9; (95%CI: 0.9–4.1); *p* = 0.080) (Table 4 and Figure 3). Among patients with short-term IUC: female gender (OR: 2.3; (95%CI: 1.3–3.9); *p* = 0.002), the catheter bag not hanging freely (OR: 0.4; (95%CI: 0.2–0.8); *p* < 0.001) and residing outside Mwanza region (OR: 0.3; (95%CI: 0.2–0.5); *p* < 0.001) were significantly associated with complications (Table 4).

## 4. Discussion

Urinary tract infections are some of the most common conditions in human medicine, affecting a large patient population to various extents, irrespective of age and gender. Community-acquired and nosocomial UTI should be considered as an essential factor of morbidity, a severe public health issue and an economic burden. The causative agents of UTI are diverse, especially in nosocomial settings (where prolonged catheterization and immunosuppression facilitate the occurrence of non-conventional urinary pathogens). These are sufficient reasons to report the prevalence and bacterial strains of UTIs on a regional basis. The article is the first to report such information for a large population.

The majority of participants enrolled in this study were married, males and significantly more among those with long-term (out-patients) IUC than with short term. For those with short-term catheterization, their catheters were removed immediately when the indication was resolved. This practice is well supported by evidence-based practice, which recommends removing the catheter as soon as possible when no longer needed [35,36], and thus prevents CA-UTI. Significantly, participants with long-term IUC were older than those with short-term IUC. This is due to the underlying physiological changes in the prostate gland that occur in older men, as is evidenced in this study, which shows that the majority had benign prostatic hypertrophy; they were elderly and above 60 years old, and had long-term catheterization, which subjected them to develop UTI. Furthermore, this study found that formal education level (i.e., patients who attained no formal education and/or primary education) was associated with the development of complications among participants with long-term IUC. This observation was also reported in previous studies [1,6,32,37,38]. Lack or little formal education means lacking the knowledge of general hygienic measures that aims at preventing and controlling infectious diseases, such as UTI. Other possible reasons for the higher prevalence of UTI in this group could possibly be due to the frequent visit to the health care setting, due to the complications the individuals were experiencing at home, such as blockage, leakage and bleeding, which predisposed them to a higher risk of developing CA-UTI. As documented by Gould et al. and Shiralizadeh et al., frequent clinics or hospital visits and hospitalization are among the factors reported to increase the risks of CA-UTI [2,16].

We also found that patients with long-term catheterization of ≥6 weeks had a two-fold risk of developing complications and as the duration in days increased, the risk of complications was also found to increase almost four times and it was statistically significant. Studies also report that the longer the catheter remains in situ, the higher the rate of bacteria colonizing the catheter bag and ascending in the drainage tubing towards the urinary bladder, resulting in CA-UTI [6,39]. Regarding the instructions on living with a catheter at home, the majority of the participants in this study were found to have not been instructed on how to manage their catheters at home and it was not statistically significant. However, according to evidence-based guidelines on good urological care practice, it is imperative that the patients and carers should be educated about catheters and the different types available, and be provided with information on all aspects of living with a catheter, from the mechanics of how it works, how to self-care and follow a hygienic regimen and prevent urinary tract infections (UTIs), blockages and leakages, to its effect on body image and maintaining normal daily life [35,40,41]. In regard to the catheter size, the majority of the study participants used size 16 Fr and it was noted to be statistically significant. However, on factors associated with complications, there was no statistical significance on the catheter size. Nevertheless, evidence-based practice recommends using the smallest size catheter 14–6 Fr that provides good drainage with a 5 mL balloon inflated with 10 cc sterile water to ensure balloon symmetry for easy drainage. Large catheters (>16 F) distend the urethra and can irreparably damage the urethra and bladder neck, as well as contribute to bladder spasms and leakage. Additionally, large bore catheters do not allow for the drainage of the peri-urethral glands, which can increase the risk of infection [41]. Therefore, with all of these observed factors, it is not surprising that out-patient participants had a high prevalence rate of CA-UTI 82.2% vs. 35.3% for the in-patients. The overall prevalence of CA-UTI in this study, which considered older participants, was higher than the results obtained in a study from Benin West Africa that enrolled younger participants between the ages of 50 to 69 who were catheterized, which observed a prevalence of 23.3% [42]. This may explain why we observed a higher prevalence of CA-UTI than the study conducted in Benin. However, our prevalence of CA-UTI is more or less equal to a study from Nigeria, which observed a prevalence of 88.5%.

On the other hand, for the in-patients of a female gender, as reported previously [25,29], a catheter bag not hanging freely and residing outside Mwanza were statistically found to be the risk factors for developing complications among participants with short-term IUC. The anatomy of women facilitates the development of UTI as bacteria have to travel a very short distance from the anus (the microbiota of the gastrointestinal tract) to reach and infect the bladder, especially if hygienic measures are not observed. A catheter bag not hanging freely was documented to be poor urological practice, which leads to acquiring UTIs. An example of the good practice of urological care, as recommended by the guidelines on the prevention of CAT-UTI, is keeping the collection bag below the level of the bladder at all times and not to rest the bag on the floor [2,35].

As in previous studies, catheter-associated urinary tract infection (CA-UTI) was also the most common complications among patients with IUC, 56.8% [37]. In this study, the other complications that were reported and/or observed were leakage, blockage and bleeding, as reported elsewhere [37,43,44]. Leakage (11.9% vs. 0.8%) and blockage (10.4% vs. 0.0%) were the more frequently observed complications, compared to the others, and were significantly higher among patients with long-term IUC compared to those with short-term IUC (*p* = 0.005). Our findings were lower compared to those by Wilde et al., who reported blockage and leakage among 34% and 67% of patients with long-term IUC, respectively, over a 12 month period of follow-up [45]. One half (56.8%) of the patients with IUC had laboratory-confirmed CA-UTI, which was significantly higher among patients with long-term catheterizations. As explained above, the longer the urinary catheter remains in situ, the higher the risk of CA-UTI from bacteria colonizing the urinary catheter bag. The prevalence of confirmed CA-UTI was significantly high compared to the prevalence in higher-income countries (HICs), the U.K. (4.76%) [46], Columbia (1.41%) [29], Australia (0.9%) [27], and England (8.5%) [47]. This could be attributed to the out-patients who had IUCs for a long-term period (≥6 weeks) outside healthcare facilities with no instructions on the proper handling of catheters at home, and infrequently changing the catheters. Catheter management in developed countries is mainly geared toward avoiding breaches in the drainage system by an adherence to a sterile closed method of urinary drainage and the maintenance of routine hygienic practices concerning the insertion site, including the use of warm soapy water, and more frequently if there is a build-up of secretions and the removal of the catheter if it is no longer needed. This practice is evident and has been shown to markedly reduce the risk of acquiring a catheter-associated infection [48]. In this study, the majority of the participants were observed to have very dirty catheter insertion sites with very hardened built-up secretions leading to UTIs.

The prevalence of CA-UTI among in-patients only in our study remains high, compared to the studies mentioned earlier—in the U.K., Columbia, Australia and England, which only enrolled in-patients. This may be due to under-improved healthcare services, notably infection prevention and control (IPC) measures in the majority of healthcare facilities in LMICs compared to HICs. Moreover, limited evidence-based literatures from LMICs limit strategies aiming at improving the lives of out-patients with IUC and healthcare services, including IPC measures among in-patients with IUC.

The majority of bacteria causing CA-UTI among patients with IUC were Gram-negative rods (GNRs) with the predominance of *E. coli* and *Klebsiella* spp. Our findings are similar to those found in the literature [12,49], which determined that GNR, notably *E. coli* and *Klebsiella* spp., are the most common causative agents of CA-UTI. These bacteria are usually from the patient’s own colonic resident microbiota with virulence factors associated with adhesion, motility, immune avoidance and biofilm formation [12,13,42]. These findings were also noted in a study conducted by Petronio et al., which determined that uropathogenic *Escherichia coli* (UPEC) is a heterogeneous group of extra intestinal pathogenic *E. coli* with several virulence factors that allow these bacteria to colonize the urinary tract and resist the host’s defense mechanisms. These virulence factors are distinctive and very different from those observed in other human microbial communities; among them are type 1 fimbriae and P fimbriae, which are present on the bacteria surface [50]. Poor hygiene on the insertion site was observed in most of the study participants, as there was a large build-up of secretions observed during sampling leading to CA-UTI

In general, GNB causing CA-UTI among patients with IUC showed higher and moderate resistance towards antibiotics of the first (i.e., ampicillin and trimethoprim-sulfamethoxazole) and second (i.e., ceftriaxone) lines of treatment. This was also previously reported by the same facility, which determined that clinical isolates exhibit a high resistance against prophylactic antibiotics and those commonly administered empirically as first and second lines of treatments [51,52]. Furthermore, a similar study by Maharjan et al. observed a higher resistance of bacteria causing CA-UTI towards ampicillin, trimethoprim-sulfamethoxazole and cephalosporin [53]. We also observed that bacteria pathogens causing CA-UTI among in-patients were more resistant to tested antibiotics, compared to bacteria pathogens causing CA-UTI among out-patients. These findings are similar to the study conducted by Gajdács et al., whereby they found that the resistance in *Klebsiella* species to 3rd generation cephalosporins (e.g., ceftriaxone) had the lowest levels of resistance, at around 15%, in the out-patient group and close to 40% in the in-patient group [54]. In our study, this may be attributed to the high empirical use of antibiotics in healthcare facilities, compared to the community use of antibiotics.

Additionally, the proportion of *E. coli* (50.6%) and *Klebsiella* spp. (47.4%) producing extended spectrum β-lactamase enzymes causing CA-UTI among patients with IUC is high. The literature reports that about 60–80% of patients with IUC receive systemic antibiotics for indications far more from UTI, resulting in the infection of the bladder with bacteria resistant to multiple antibiotics [52,55]. This observation calls for the inexpensive strategy to combat antimicrobial resistance at this facility and elsewhere, which is antimicrobial stewardship. A study conducted in Saudi Arabia [56] reported a prevalence of 9.6% of ESBL producing Gram-negative bacteria among patients with IUC. *E. coli* accounted for 11.3%, whereas *K. pneumoniae* accounted for 10.1% of ESBL production. These findings are lower compared to what has been observed in our study. This could possibly be explained by a lack of stewardship on antibiotics use on our premises.

## 5. Conclusions

This is the first study in Sub-Saharan Africa, particularly in northwestern Tanzania, which has shown the prevalence CA-UTI among out-patients and in-patients with an indwelling urinary catheter. *E. coli* and *K. pneumoniae* are the common pathogens causing CA-UTI among out-patients with IUC than in-patients with IUC. Moreover, pathogens causing CA-UTI among in-patients were more resistant to antibiotics than those causing CA-UTI among out-patients. Out-patients with IUC were more likely to develop CA-UTI than in-patients, which could also affect their overall well-being. Different factors, including the duration of catheterization, were found to predict CA-UTI. We therefore recommend the educating of patients and carers on how to manage their catheters, maintain hygiene and perform self-care, and early surgical intervention to correct the indications leading to prolonged catheterization, and thus reduce CA-UTI among patients living at home with indwelling catheters.

## Figures and Tables

**Table 1:** Socio-demographic characteristics of participants with short-term IUC (in-patients) and long-term IUC (out-patients).

Patient Characteristics	Out-Patients (*N* = 202), *n* (%)	In-Patients (*N* = 238), *n* (%)	*p*-Value
***Median age [IQR] in years***	69 (61–77)	46 (30–62)	<0.001
***Sex***			
Males	194 (96.0)	132 (55.5)	<0.001
Females	8 (4.0)	106 (44.5)	
***Residence***			
Outside Mwanza	117 (57.9)	119 (50)	0.097
Mwanza	85 (42.1)	119 (50)	
***Marital status***			
Married	187 (92.6)	195 (81.3)	<0.001
Single	15 (7.4)	43 (18.1)	
***Occupation***			
Peasants	136 (67.3)	164 (68.9)	<0.001
Retired	29 (14.4)	2 (0.8)	
Petty traders	26 (12.9)	64 (26.9)	
Civil servants	11 (5.5)	8 (3.4)	
***Religion***			
Christians	153 (75.7)	202 (84.9)	0.041
Muslims	26 (12.9)	22 (9.2)	
Others	23 (11.4)	14 (6.0)	
***Education***			
No formal education	43 (21.3)	17 (7.1)	<0.001
Primary level	116 (57.3)	198 (83.2)	
Secondary level	29 (14.4)	18 (7.6)	
College and above	14 (6.9)	5 (2.1)	

Key: IQR = Interquartile range.

**Table 2:** Clinical characteristics of participants with short-term IUC (in-patients) and long-term IUC (out-patients).

Patient Characteristics	Out-Patients (*N* = 202), *n* (%)	In-Patients (*N* = 238), *n* (%)	*p*-Value
***Type of catheter***			
Supra-pubic type	82 (40.6)	10 (4.2)	<0.001
Urethral type	120 (59.4)	228 (95.8)	
***Catheter size (FR)***			
14 FR	19 (9.4)	70 (29.4)	<0.001
16 FR	158 (78.2)	149 (62.6)	
18 FR	15 (7.4)	19 (8.0)	
20 FR	10 (5.0)	-	
***Instructions for proper handling of catheters at home for out-patients***			
Given	20 (9.9)	NA	NA
Not given	182 (90.1)	NA	
***Catheter change for out-patients***			
Changed	123 (61.0)	NA	NA
Not changed	79 (39.1)	NA	
***Duration of catheter* in situ *(weeks)***			
0–2	0 (0.00)	238 (100)	<0.001
3–5	91 (45.1)	0 (0.00)	
≥6	111 (55.0)	0 (0.00)	
***Comorbidity***			
With comorbidity	44 (21.8)	48 (20.2)	0.163
No comorbidity	158 (78.2)	190 (79.8)	
***Indications for catheterization***			
BPH	122 (60.4)	9 (3.8)	<0.001
Urethral stricture	37 (18.3)	19 (8.0)	
Urine retention	24 (11.9)	-	
Urinary incontinence	13 (6.4)	78 (32.8)	
CNS	2 (0.9)	24 (10.1)	
Fistula	-	25 (10.5)	
Others	29 (14.4)	79 (33.2)	
***Complications associated with IUC***			
CA-UTI	108 (53.5)	78 (32.7)	0.005
Leakage and CA-UTI	25 (11.9)	2 (0.8)	
Blockage and CA-UTI	21 (10.4)	-	
Bleeding and CA-UTI	12 (5.4)	4 (1.7)	
Leakage of urine	3 (1.5)	4 (1.7)	
Blockage of catheter	1 (0.5)	1 (0.4)	
Bleeding	-	1 (0.4)	
None	34 (16.8)	149 (62.6)	

Key: FR = French size; BPH = benign prostatic hypertrophy; CNS = central nervous system; CA-UTI = catheter-associate urinary tract infection.

**Table 3:** Percentage resistance of bacteria causing CA-UTI among patients with short-term and long-term IUC.

Bacterial Species	AMP *n* (R%)	SXT *n* (R%)	CN *n* (R%)	CIP *n* (R%)	AMC *n* (R%)	CRO *n* (R%)	CAZ *n* (R%)	F *n* (R%)	MEM *n* (R%)	ESBL *n* (%)	
										Positive	*p*-Value
***E. coli***											
Out-patients (55)	52 (94.5)	46 (83.6)	20 (36.4)	24 (43.6)	41 (74.5)	25 (45.5)	27 (49.1)	17 (30.9)	14 (25.4)	25 (45.5)	0.6013
In-patients (24)	23 (95.8)	21 (87.5)	14 (58.3)	16 (66.7)	22 (91.7)	12 (50.0)	13 (54.2)	11 (45.8)	0 (0.0)	12 (50.0)	
All (79)	75 (94.6)	67 (84.8)	34 (43.0)	40 (50.6)	63 (79.7)	37 (46.8)	40 (50.6)	28 (35.4)	14 (17.7)	37 (50.6)	-
***Klebsiella* spp.**											
Out-patients (47)	47 (100)	38 (80.9)	11 (23.4)	13 (27.7)	38 (80.8)	19 (40.4)	18 (38.3)	20 (42.6)	12 (25.5)	18 (38.3)	0.0766
In-patients (29)	29 (100)	25 (86.2)	13 (44.8)	12 (41.4)	23 (79.3)	17 (58.6)	15 (51.7)	15 (51.7)	0 (0.0)	18 (62.1)	
All (76)	76 (100)	63 (82.9)	24 (31.6)	25 (32.9)	61 (80.3)	36 (47.4)	33 (43.4)	35 (46.1)	12 (15.7)	36 (47.4)	-
***Other Gram-negative rods (GNR)***											
Out-patients (69) *	46 (80.7)	34 (59.6)	14 (20.3)	22 (31.9)	56 (81.3)	19 (33.3)	23 (33.3)	31 (44.9)	19 (27.5)	NT	NA
In-patients (30) **	11 (52.4)	9 (42.9)	6 (20.0)	9 (30.0)	23 (76.4)	7 (33.3)	6 (20.0)	9 (30.0)	0 (0.0)	NT	NA
All GNR (99) ***	57 (73.1)	43 (55.1)	20 (20.2)	31 (31.3)	79 (79.6)	26 (33.3)	29 (29.3)	40 (40.4)	19 (19.2)	NT	NA

Key: NT = not tested; NA = not applicable; AMP = ampicillin; SXT = trimethoprim-sulfamethoxazole; CIP = ciprofloxacin; CRO = ceftriaxone; CAZ = ceftazidime; F = nitrofurantoin; and ESBL = extended spectrum β-lactamase. For GNR, the denominators were 57 (*), 21 (**) and 78 (***) for out-patients, in-patients and all GNR, respectively. For the AMP, SXT, AMC and CRO, the antibiotic discs were not included in the antimicrobial susceptibility testing of *P. aeruginosa* (*N* = 21, out-patients *n* = 12 and in-patients *n* = 9).

**Table 4:** Factors associated with complications among patients with short-term and long-term IUC.

Patients’ Characteristics		Patients with Long-Term IUC (Out-Patients)				Patients with Short-Term IUC (In-Patients)			
		Complications		Analysis		Complications		Analysis	
		YES *n* (%)	NO *n* (%)	OR [95%CI]	*p*-Value	YES *n* (%)	NO *n* (%)	OR [9 5%CI]	*p*-Value
Age in years	69 (61–77) years	168 (83.2)	34 (16.8)	0.99 [0.96–1.0]	0.341	NA	NA	NA	NA
	46 (30–62) years	NA	NA	NA	NA	89 (37.4	149 (62.6)	0.9 [0.98–1.0]	0.309
Gender	Males	162 (83.5)	32 (16.5)	1.0	0.533	38 (28.8)	94 (71.2)	1.0	0.002
	Females	6 (75)	2 (25.0)	0.6 [0.1–3.1]		51 (48.1)	55 (51.9)	2.3 [1.3–3.9]	
Marital status	Single	13 (86.7)	2 (13.3)	1.0	0.708	17 (39.5)	26 (60.5)	1.0	0.749
	Married	155 (82.9)	32 (17.1)	0.7 [0.2–3.5]		72 (36.9)	123 (63.1)	0.9 [0.5–1.8]	
Education level	No formal education	31 (72.1)	12 (27.9)	1.0	0.029	12 (70.6)	5 (29.4)	1.0	0.092
	Primary	97 (83.6)	19 (16.4)	1.8 [1.1–3.1]		68 (34.3)	130 (65.7)	0.6 [0.3–1.1]	
	Secondary	28 (96.6)	1 (3.5)			8 (44.4)	10 (55.6)		
	College	12 (85.7)	2 (14.3)			1 (20.0)	4 (80.4)		
Comorbidity	HIV/AIDS	3 (100)	0 (0.00)	1.0	0.757	2 (22.2)	7 (77.8)	1.0	0.408
	DM	6 (100)	0 (0.00)	0.99 [0.99–1.0]		1 (14.3)	6 (85.7)	0.9 [0.9–1.0]	
	HTN	13 (81.3)	3 (18.8)			1 (8.3)	11 (91.7)		
	Anemia	4 (66.7)	2 (33.3)			8 (47.1)	9 (52.9)		
	Others	33 (84.6)	6 (15.4)			22 (57.9)	16 (42.1)		
	None	109 (82.6)	23 (17.4)			55 (35.5)	100 (64.5)		
Duration of catheter	<2 weeks	NA	NA	NA	NA	89 (37.4)	149 (62.6)	Collinearity	NA
	3–5 weeks	71 (78.0)	20 (22)	1.0	0.080	NA	NA	NA	NA
	≥6 weeks	97 (87.4)	14 (12.6)	1.9 [0.9–4.1]		NA	NA	NA	NA
Type of catheter	Urethral	101 (84.2)	19 (15.8)	1.0	0.647	84 (36.8)	144 (63.2)	1.0	0.405
	Supra-pubic	67 (81.7)	15 (18.3)	1.2 [0.6–2.5]		5 (50.0)	5 (50.0)	0.6 [0.2–2.1]	
Catheter size	14 FR	17 (89.5)	2 (10.5)	1.0	0.923	31 (44.3)	39 (55.7)	1.0	0.167
	16 FR	130 (82.3)	28 (17.7)	1.0 [0.5–1.8]		52 (34.9)	97 (65.1)	0.7 [0.5–1.1]	
	18 FR	12 (80)	3 (20)			6 (31.6)	13 (68.4)		
	20–26 FR	9 (90.0)	1 (10.0)			NA	NA		
Indication for catheterization	BPH	98 (80.3)	24 (19.7)	1.0	0.539	2 (22.2)	7 (77.8)	1.0	0.145
	Urethral stricture	33 (89.2)	4 (10.8)	1.1 [0.8–1.4]		6 (31.6)	13 (68.4)	1.1 [0.9–1.3]	
	Urinary incontinence	12 (92.3)	1 (7.7)			18 (23.1)	60 (76.9)		
	CNS/unconsciousness	0 (0.00)	1 (100)			11 (39.3)	17 (60.7)		
	Fistula	-	-			23 (92.0)	2 (8.0)		
	Others	25 (86.2)	4 (13.8)			29 (36.7)	50 (63.3)		
Instructions on living with catheter at home	Not given	116 (63.7)	66 (36.3)	1.0	0.110	NA	NA	NA	NA
	Given	11 (55.0)	9 (45.0)	0.5 [0.2–1.2]		NA	NA	NA	
Residence	Mwanza	72 (84.7)	13 (15.3)	1.0	0.619	29 (24.4)	90 (75.6)	1.0	<0.001
	Outside Mwanza	96 (82.0)	21 (17.9)	1.2 [0.6–2.6]		60 (50.4)	59 (49.6)	0.3 [0.2–0.5]	
Occupations	Retired	23 (79.3)	6 (20.7)	1.0	0.399	1 (50.0)	1 (50.0)	1.0	0.539
	Peasants	112 (82.4)	24 (17.6)	1.2 [0.8–2.0]		63 (38.4)	101 (61.6)	0.9 [0.7–1.2]	
	Civil servants	11 (100)	0 (0.00)			3 (37.5)	5 (62.5)		
	Petty traders	22 (84.6)	4 (15.4)			22 (34.4)	42 (65.6)		
Positioning of urine bags	Hanging freely	NA	NA	NA	NA	45 (29.4)	108 (70.6)	1.0	<0.001
	Not hanging freely	NA	NA	NA		44 (51.8)	41 (48.2)	0.4 [0.2–0.8]	
Wards	Medical	NA	NA	NA	NA	10 (23.3)	33 (76.7)	1.0	0.644
	Surgical	NA	NA	NA		65 (44.2)	82 (55.8)	1.1 [0.7–1.7]	
	Others	NA	NA	NA		14 (29.2)	34 (70.8)		

## Data Availability

The data presented in this study are available on request from the corresponding author.
